# A STEPwise Approach for Oral Hygiene Behavior of Schoolchildren in Romania

**DOI:** 10.3390/healthcare12020198

**Published:** 2024-01-14

**Authors:** Anca-Cristina Perpelea, Ruxandra Sfeatcu, Mihaela Tănase, Marina Meleșcanu Imre, Alexandra Ripszky Totan, Ana Cernega, Cristian Funieru, Silviu-Mirel Pițuru

**Affiliations:** 1 Department of Organization, Professional Legislation and Management of the Dental Office, Faculty of Dentistry, “Carol Davila” University of Medicine and Pharmacy, 17-23 Plevnei Street, 020021 Bucharest, Romania; ana.cernega@umfcd.ro (A.C.); silviu.pituru@umfcd.ro (S.-M.P.); 2 Oral Health and Community Dentistry Department, Faculty of Dentistry, “Carol Davila” University of Medicine and Pharmacy, 17-21 Calea Plevnei Street, 010221 Bucharest, Romania; 3 Department of Pedodontics, Faculty of Dentistry, “Carol Davila” University of Medicine and Pharmacy, 17-21 Calea Plevnei Street, 010221 Bucharest, Romania; mihaela.tanase@umfcd.ro; 4 Department of Prosthodontics, Faculty of Dentistry, “Carol Davila” University of Medicine and Pharmacy, 17-23 Calea Plevnei, 010221 Bucharest, Romania; marina.imre@umfcd.ro; 5 Department of Biochemistry, Faculty of Dentistry, “Carol Davila” University of Medicine and Pharmacy, 17-23 Plevnei Street, 020021 Bucharest, Romania; alexandra.ripszky@umfcd.ro; 6 Department of Preventive Dentistry, Faculty of Dentistry, “Carol Davila” University of Medicine and Pharmacy, 17-23 Plevnei Street, 020021 Bucharest, Romania; cristian.funieru@umfcd.ro

**Keywords:** oral health, STEPS approach, oral habits, dental hygiene, fluoride toothpaste, family, schoolchildren, education

## Abstract

This research analyzes the dental hygiene habits of schoolchildren from parents’ perspectives, using the STEPS approach recommended by the World Health Organization. The key points of oral health care in children include the role of the family in encouraging and maintaining proper oral hygiene practices. This study aimed to assess the oral hygiene practices of schoolchildren with on-site dentists in Romania. Additionally, it sought to establish correlations between these behaviors and the educational levels of the adults with whom they live with. The participants were selected from the zero grade to the eighth grade, totaling 3843students. Statistical analysis involved the application of Fisher’s Exact Test and Z-tests with Bonferroni correction. Multinominal and binominal logistic regression models were employed to predict the impact of parents’ education on children’s oral health status and behavior. The oral health status of children evaluated by parents as poor was more frequent in children whose parents had a primary or gymnasium education (*p* < 0.001). Female adults with a university education evaluated the gum health of their children as very good to a much greater extent (41.7%, *p* < 0.001). Regarding the use of auxiliary means, the majority of parents with a university education mentioned that their children use dental floss (26.4% females/27.4% males) (*p* < 0.001), compared to those with primary education, where the percentage was only (1.2% males/3.5% females) (*p* < 0.001). The results of this study highlight that the education level of the adults with whom the children live with influences the perception of the teeth and gums health status, the frequency of oral hygiene, the use of fluoride toothpaste, and auxiliary brushing aids.

## 1. Introduction

The state of oral health is influenced by oral conditions that cause discomfort or tooth loss, having a negative impact on the appearance, quality of life, or nutritional intake influencing the growth and development of children. Caries and gum disease are the most widespread health problems, affecting more than 80% of children in some countries [1]. Oral conditions limit participation in school, professional, or domestic activities, leading to absences from school or work, which causes a significant loss of school or work hours globally. Moreover, the psychosocial impact of these problems negatively affects the quality of life [2].

It is useful to manage the factors that have an impact on children’s oral health in order to develop and implement supporting public health actions focused on children and parenting behaviors in an effort to provide them optimal oral care and a better quality of life [3]. Schools offer a favorable framework for promoting oral health because, in the world, more than a billion children are enrolled in educational institutions. School health education programs can also contribute to the improvement and well-being of families, school staff, and community members [1].

It is universally accepted that socioeconomic level influences health status [4]. This also applies to oral health [5]. Economic standing is associated with a higher risk of carious lesions [6]. Educating parents and children about tooth decay prevention has long been considered a fundamental element of any dental treatment plan [7]. That is why parents’ knowledge of oral health and appropriate oral health care practices for children are very important [8]. The increased incidence of dental caries is not solely determined by biological factors interacting with the causative microorganisms. Carious lesions are also associated with socioeconomic conditions, education, and eating habits [9].

It has been shown that the incidence of carious lesions is higher in disadvantaged cities in certain countries [10,11]. Key aspects of oral health care in pediatric patients include factors that highlight a family’s ability to promote and maintain appropriate oral hygiene behaviors. Parents with poor oral hygiene habits are risk factors for dental caries in their children [12].

In addition to this determinants, oral health is related to lifestyle, which is an important factor in most chronic diseases. A protective element of oral health is proper oral hygiene and age-appropriate exposure to fluoride [2]. The effects of fluoride on the prevalence of dental caries incidence is confirmed, and the correlation with socio-economic status is validated [13]. The most significant way to benefit from the positive effects of fluoride is the use of fluoride-containing toothpaste [14]. Numerous studies support the promotion of tooth brushing with fluoridated toothpaste in the context of oral health programs conducted in schools [15,16,17].

Drinking water, beverages that are made with fluoridated water, and certain foods are major sources of fluoride in general. The American Dental Association suggests an ideal concentration of 0.7 ppm fluoride, equivalent to 0.7 mg fluoride per liter of water, in drinking water [18]. Since, in general, drinking water sources in Romania are low in fluoride, the probability of suffering an overdose through the use of topical treatments is minimal [19]. Therefore, it is imperative to assess exposure to risk factors using appropriate surveys to identify the vulnerable population, as well as the behaviors with the highest risk potential that require addressing. This must be performed before planning and implementing oral health promotion programs for children [20]. There is a lack of centralized national studies providing information on the oral health status of schoolchildren in Romania [21]. Information regarding oral health education represents the means by which a shift in dental treatments can be achieved, moving from invasive therapy to prevention [21].

In Romania, the dental care system is both public and private. According to data provided by National Institute of Public Health, in the year 2021, approximately 20,000 dentists were registered in Romania, with around 20% of them working in the public system. Regarding educational institutions, only 3% of the educational units have a school office and a dentist [22]. This percentage is too small to ensure preventive, diagnostic, and treatment dental procedures for the school population.

The present study aimed to carry out a detailed analysis of the oral hygiene behavior of students in relation to the level of education of the adult with whom the child lives and the family’s living environment.

## 2. Materials and Methods

### 2.1. Study Design and Sample Selection of Participants

The educational system in Romania is organized into 9 educational levels, called the International Standard Classification of Education (ISCED 0 to 8): early education (ISCED 0), primary education (ISCED 1), lower secondary education (ISCED 2), upper secondary education (ISCED 3), non-university tertiary education (ISCED 4), higher education (ISCED 5–8) [23].

**Figure 1:**
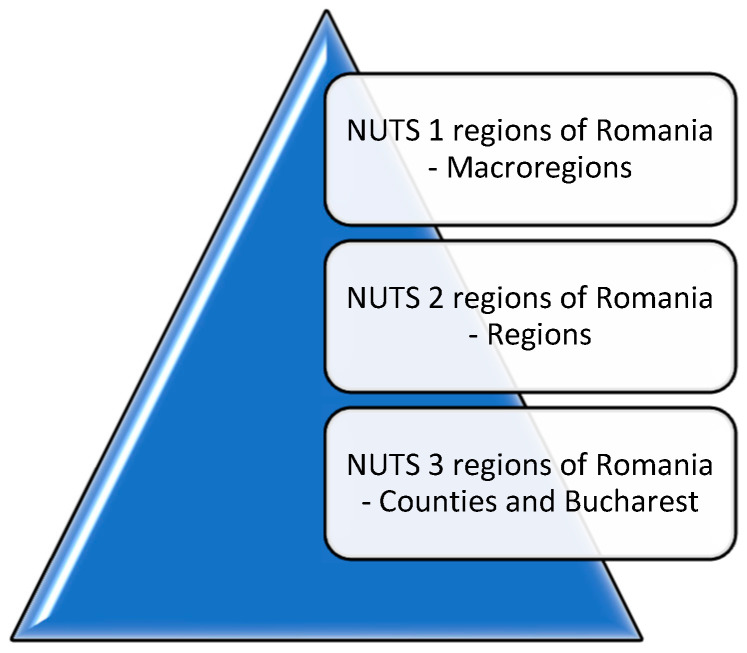
Nomenclature of Territorial Units for Statistics (NUTS)—the levels for Romania.

In this study, carried out in the period 2022–2023, students enrolled in public educational institutions, from the educational level ISCED level 1 (grade 0 to grade 4) and ISCED level 2 (grade 5 to grade 8), were selected. Students were selected from 35 counties (NUTS 3) and the 6 sectors of Bucharest (capital of the country), from schools where there is a dental practice authorized and school dentist, according to the methodology developed by the National Institute of Public Health in Romania (Figure 1) [24].

A minimum of 2 schools were selected from each county and from each sector of Bucharest and a minimum of 5 children from each age category. In accordance with Romanian regulations, children are enrolled in the school unit in the family’s area of residence, but there are also situations in which parents can choose another educational unit outside the school district [25]. According to the situation analysis carried out by the National Institute of Public Health, in Romania there are 467 school dental offices in the urban environment [22], and Bucharest is the most populated city in Romania with 137 school dental offices [26]. The participants were selected from grade 0tograde 8, with a total of 3843 students, respectively, parents/legal representatives who completed the informed consent form for participation of the student in the study, as well as the evaluation questionnaire related to the oral health status of the child. For children 7 years to 14 years, the age distribution was relatively homogeneous, the most frequent age categories being 9 years (11.9%) and 10 years (11.9%). The mean age was 10.56 ± 2.61 years, the median being 11 years (interpercentile range: 8–13 years); 53.4% are females.

### 2.2. Ethical Consideration

Before implementation, the study was submitted to the Research Ethics Committee of the “Carol Davila” University of Medicine and Pharmacy Bucharest. It was approved in accordance with the Methodology for Monitoring Oral Health in Schools, having the registration number 36927/29.11.2022. Written consent was obtained from the legal representative of the children participating in the study.

### 2.3. The Translation and the Adaptation of the Questionnaire

The data were obtained by applying a questionnaire dedicated to children, developed by the World Health Organization (WHO) and presented within the methodology published in 2013 [27]. According to the methodology of the present study, the self-administered questionnaire was filled out by the parents of the students who previously completed the informed consent form regarding the child’s participation in the study. Prior to application, the STEPS questionnaire was validated and adapted to the target group, the parents of students in grades 0–8, according to the WHO methodology from 2020 [28]. In this sense, the English version of the questionnaire was translated into Romanian in the first stage by two bilingual Romanian translators, then the two translations were compared in a face-to-face meeting, where the authors of the study consulted with two specialists in education and sociology and made sure that the way each question was formulated did not change the meaning of the answer options. The retroversion was carried out by an independent translator, from Romanian to English, then the version administered to the respondents was completed. This version was tested among 20 adults who were asked to rate the clarity and difficulty of the questions and answers. This did not lead to the need for any changes. The questionnaire consisted of items related to: general information (age and gender of the child, class, as well as socio-demographic data, background and level of education of the parents); parents’ perception of their children’s oral health (teeth and gums) and information related to their children’s personal oral hygiene; frequency of tooth brushing; means used for oral hygiene; including auxiliary aids; use of toothpaste; parents’ knowledge of using fluoride toothpaste.

## 3. Results

**Figure 2:**
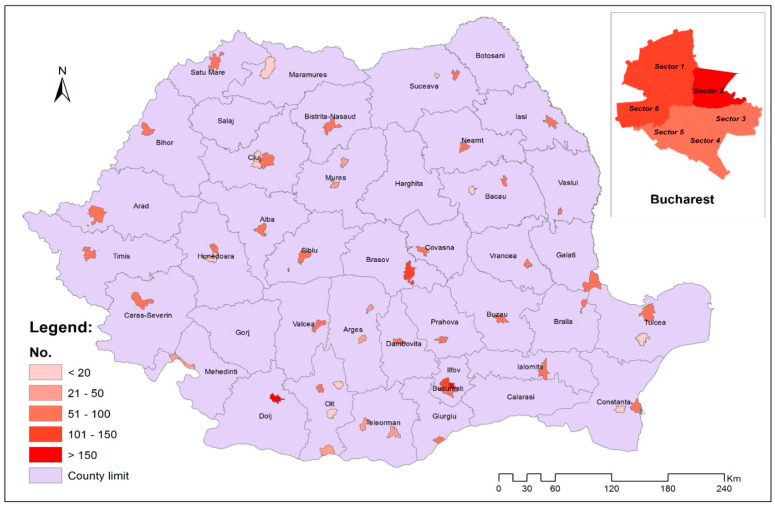
The territorial distribution of the studied sample by counties (NUTS 3).

The distribution of the participants analyzed in the study related to the children’s place of origin is presented in Figure 2. Most children come from Bucharest (16.81%), and a percentage of 89.5% reside in the urban environment.

Among the parents of the children participating in the study, and out of the majority of female adults with whom they lived, 62.4%, had a university education, 29.6% ahigh school education, 4.8% a secondary school education, 0.4% of the children did not live with a female adult, and 206 did not answer this question. According to the results, the majority of male adults had a high school education (35.6%) or a university education (51.3%), 5.5% of children did not live with a male adult, and 216 preferred not to answer.

The statistical analysis was performed using IBM SPSS Statistics 25 and illustrated using Microsoft Office Excel/Word 2021. Qualitative variables were expressed in absolute terms or as percentages, and they were tested between groups using Fisher’s Exact Test. Z-tests with Bonferroni correction were conducted to further detail the results obtained in contingency table.

Very good health status was significantly more common in children whose male adults had a university education (33.2%) compared to those with a high school/gymnasium or primary education. Health status perceived as very good was significantly more frequent in children whose adults, both female and male, had a university education rather than high school or gymnasium. Excellent health status was significantly more common in children whose female adults had a university education compared to those with a high school education.

The poor oral health status of children’s teeth assessed by parents was significantly more frequent in children whose parents have primary or gymnasium education (*p* < 0.001) (Table 1).

The perception of gum health as poor was significantly more frequent in children whose male adults had a gymnasium education (9.2%), than a high school or university education (1.9%/0.6%).

Female adults with a university education rated the health status of their children’s gums as very good in a much higher proportion (41.7%, *p* < 0.001), compared to those with a high school or gymnasium education (Table 2).

Regarding differences in gingival health, it has been noted that very good status was significantly more common in children whose male adults had a university education (42.1%), compared to those with high school, gymnasium, or primary education. Excellent gum status was significantly more common in children whose male adults had a university education.

Regarding the frequency of dental hygiene among children, most parents who mentioned that their children brush at least twice a day were those with university education. Absent dental hygiene was significantly more frequent in children whose adults had a primary education (Table 3).

Among the disparities noted regarding the frequency of dental hygiene, it has been noticed that dental hygiene practiced two or more times a day was significantly more common in children whose male adults had a university education (58.9%), compared to those with gymnasium (29.7%) or primary education (17.4%). It has been noted that dental hygiene practiced several times a week was significantly more common in children whose female adults had a primary education (15.1%) compared to those with an academic education. Similarly, the case of tooth brushing once a day was higher among children with high school educated female adults (44.3%).

The behavior of the parents regarding the use of toothpaste and fluoride toothpaste is described in Table 4; it was observed that the higher the level of education of the adult with whom the child lives, greater attention is paid to the use of toothpaste and the use of fluoride toothpaste (*p* < 0.001) respectively. 

The results show that the use of fluoride toothpaste was significantly more common in children whose male adults had a university/high school/gymnasium education (79.9%/75.2%/68.9%), compared to those with a primary education (41.8%). In the case of female adults who responded to this question, the absence of fluoride toothpaste usage was significantly more common in children whose female adults had a primary education (57.7%), compared to those with a gymnasium/high school/university education (31.5%/27.2%/19.9%).

Regarding the use of auxiliary means by children, the majority of parents with a university education mentioned that their children use dental floss (26.4% female/27.4% male), compared to those with a primary education, where the percentage was only 3.5% female/1.2% male (*p* < 0.001) (Table 5).

In relation to predicting the perceived oral health status of teeth and gums, as well as the frequency of tooth brushing and dental hygiene auxiliary aids, the multinomial regression models were employed. For predicting the use of toothpaste, the fluoride toothpaste, toothbrush, dental floss, and plastic toothpicks, bivariate regression models were utilized (Table 6).

In the case of dental health, both univariate and multivariate models (where both parents’ education levels were simultaneously considered in the prediction model) showed that the education levels of both parents had a significant effect on perceived health and oral hygiene habits of their children. Thus, observing the increasing trends of odds ratio (OR) values, compared to non-university education, a university education increase the odds of having a better perceived health status. The increase in odds is more pronounced for very good or excellent oral health states compared to others (comparing OR values among themselves). In the case of gum health, in the multivariable model, only the mothers’ education had a significant effect, exhibiting the same trend as observed for teeth health. Regarding the frequency of dental hygiene, a similar pattern is observed; both mothers’ and fathers’ education levels have a significant effect on dental hygiene frequency, similar to teeth/gums health. The increase in the odds of having better dental hygiene due to the presence of university education is greater for the appropriate frequency of dental hygiene compared to absent/very rare hygiene (a few times a month) (Table 6).

For the use of toothpaste, fluoride toothpaste, and plastic toothpicks, according to multivariable models, only mothers’ education levels had a significant and important effect on their usage. For example, the presence of university education in mothers significantly increased (*p* = 0.001) the odds of using fluoride toothpaste for children by 1.484 times (95% confidence interval: 1.174–1.875).

## 4. Discussion

The development of behavioral habits begins in childhood, involving parents, having an essential role in the formation of behavior related to oral health care of children.

Based on the results highlighted above, the level of education of the adults with whom the children live influences six categories of factors. These elements are: the perception of children’s dental health status, the perception of children’s gingival health status, the performance of oral hygiene and its frequency, as well as the use of fluoride toothpaste and brushing aids. This aspect can mean understanding the contextual elements associated with the way of life and level of education of adults.

In accordance with the objective of the study, the influence of socioeconomic factors and the perception of oral health status were highlighted in children from grades 0–8 in Romania. The differences between the groups were not significant, so the rural residence of the children was not significantly associated with the health status or the frequency of oral hygiene. This particularity is probably determined by the predominance of the urban living environment in which the child lives and by the fact that the study took place only in the urban environment, where there were dental offices in schools.

During the school years, the identification of risk factors leading to the appearance of carious lesions can refine the classification of caries risk in students and improve the management of existing resources in order reduce oral health inequalities [29,30,31].

Scientific articles have demonstrated that parents’ level of education and their social class have a significant impact on the profile of children’s oral health status [32]. The results of their analysis show that the parent’s level of education and increased family income decreases the prevalence of dental caries. Thus, the parents’ level of education and their profession influence the children’s oral health status [33]. Similar to the research carried out in Romania, it is highlighted that the lack of education of the parents has influences on the oral hygiene behaviors of the children [34]. Socioeconomic factors have also been correlated with the use of brushing aids [35]. Similar to another study conducted in Romania, the results highlight a potential influence of the parents’ education level, both for the mother and the father, regarding the dental brushing habits of children [21].

According to a study carried out in the Netherlands, the socioeconomic level of the mother is closely related to the occurrence of dental caries. The prevalence of carious lesions being much higher in the population categories with low socioeconomic level [5]. A range of studies highlights the positive impact of school-based oral health programs, especially those based on gamification [36]. Inequalities related to the different education levels of parents can be reduced with the help of programs initiated in school. School-based oral health programs represent effective tools for eliminating disparities in oral health [37]. Targeting teachers could improve the effectiveness of dental health education campaigns for children [38]. It is emphasized that maintaining a satisfactory level of oral hygiene at home depends on parental involvement, toothbrushing instructions, and educational programs [39].

In Denmark, Christensen et al. highlighted that a low maternal education level, low family wage income, and large families are associated with an increased prevalence of carious lesions [40]. The connection between the socioeconomic level of the family and dental hygiene ishighlighted in this study, and the presence of caries is highlighted in other studies [41]. These studies should stimulate the development of effective prevention strategies, with special attention to social classes with low incomes.

Attitudes and practices related to oral hygiene, diet, and individual factors related to cariogenesis can be effectively managed by ensuring access to the resources students need, even if they are limited [34]. Understanding the evolution of the distribution of risk factors and the prevalence of carious lesions with the help of repeated surveys is crucial for making the adjustment of strategies developed at the community level for the promotion of oral health [20].

The differences between the knowledge related to the health of the stomatognathic system and the attitudes of parents regarding oral hygiene underline the importance of oral health education [42]. Since oral hygiene is an important factor for oral health, adequate guidelines on dental hygiene methods and the relationship between dental hygiene and dental caries should be provided to the population [3]. Therefore, we should investigate these behaviors in more detail in order to provide a solid basis for prevention programs [43].

Strengths: The present research represents one of the most important studies carried out in our country, being the only national study that uses the methodology developed by the National Institute of Public Health and that evaluates the influence of family socioeconomic factors and the perception of adults on the behaviors related to children’s oral hygiene. It is the first study carried out in Romania that correlates the socioeconomic status of the parents and the use of fluoride toothpaste. In contrast to the previous studies carried out in Romania, which were carried out on a smaller sample, in this national study the cohort is composed of a large number that determines a more detailed analysis and a broader understanding of the determinants involved in the analysis of oral health status. This research reinforces the need for the development of prevention strategies for oral health, among children in Romania.

Limitations of the study: The correspondence between the parents’ perception with the oral condition of the children has not been assessed. The study was carried out in the urban environment, which led to an image that did not include the situation in the rural environment, where there are no school dental offices.

Possible further directions of research are the evaluation of the students’ behaviors related to diet and pattern of dental visits, as well as the correlation with clinical evaluation of the oral status carried out by the school dentists. The relationship between the effect of rurality, the educational level of the parents, and the oral health behavior of children can also be evaluated.

## 5. Conclusion

This research demonstrates that there is a possible correlation between the education level of the adult with whom the child lives and the perceived health and oral hygiene habits of their children. Furthermore, both mothers’ and fathers’ education have an impact on the frequency of oral hygiene among children. Building on the results that emphasize the use of fluoride toothpaste, the mother’s education level had a significant influence on their usage. Therefore, the main objective of school campaigns should be to inform and raise awareness among the target population regarding the importance of performing dental toothbrushing and using auxiliary means for tooth cleaning. It would be highly beneficial for students to practice tooth-brushing techniques and to learn about auxiliary brushing tools in the school dental offices. This is because, once children have acquired these skills, they can then educate their parents about these habits at home. The findings suggest that is crucial to have educational programs in Romania for taking care of schoolchildren’s oral health that involve parents, along with educators, including teachers and professors, under the guidance of dentists.

## Figures and Tables

**Table 1:** The perception of children’s dental health related to the socioeconomic status of the parents.

**Health**	**Rural**				**Urban**					***p***
	**No.**		**%**		**No.**		**%**		**Missing**	
Poor	14		3.8%		170		5.4%		0	0.648
Satisfactory	54		14.5%		414		13.2%		0	
Good	174		46.7%		1422		45.1%		0	
Very good	100		26.9%		886		28.2%		0	
Excellent	30		8.1%		254		8.1%		0	
Missing	31		7.69% of Rural		294		8.54% of Urban		0 **	325 (8.45%) ***
**Health/** **Studies-M**	**Primary**		**Gymnasium**		**High School**		**Academic**		**Missing**	*p* *
	**No.**	**%**	**No.**	**%**	**No.**	**%**	**No.**	**%**		
Poor	13	16.9%	21	14.5%	78	6.5%	49	2.7%	23 (12.5%)	<0.001
Satisfactory	12	15.6%	16	11%	190	15.7%	177	9.8%	73 (15.6%)	
Good	37	48.0%	72	49.7%	591	48.9%	787	43.6%	109 (6.82%)	
Very good	12	15.6%	27	18.6%	280	23.2%	601	33.2%	66 (6.69%)	
Excellent	3	3.9%	9	6.2%	69	5.7%	194	10.7%	9 (3.17%)	
Missing	10	11.49%	17	10.49%	86	6.64%	54	2.9%	158 (4.11%) **	605 (15.74%) ***
**Health/** **Studies-F**	**Primary**		**Gymnasium**		**High School**		**Academic**		**Missing**	*p* *
	**No.**	**%**	**No.**	**%**	**No.**	**%**	**No.**	**%**		
Poor	8	10.7%	25	17.1%	80	8%	67	3%	4 (2.17%)	<0.001
Satisfactory	8	10.7%	22	15.1%	155	15.5%	245	11.1%	38 (8.12%)	
Good	39	52%	65	44.5%	509	51%	954	43.4%	29 (1.81%)	
Very good	17	22.6%	27	18.5%	199	20%	720	32.7%	23 (2.33%)	
Excellent	3	4%	7	4.8%	55	5.5%	215	9.8%	4 (1.4%)	
Missing	12	13.79%	30	17.04%	80	7.42%	69	3.04%	134 (3.48%)	423 (11%) ***

* Fisher’s Exact Test, ** Missing data with none of the characteristics observed, *** Total missing.

**Table 2:** Perception of oral health status of children’s gums according to parents’ socioeconomic status.

**Health**	**Rural**				**Urban**					***p***
	**No.**		**%**		**No.**		**%**		**Missing**	
Poor	9		2.6%		45		1.6%		0	0.293
Satisfactory	30		8.5%		186		6.4%		0	
Good	130		36.9%		1120		38.7%		0	
Very good	130		36.9%		1059		36.5%		0	
Excellent	53		15.1%		488		16.8%		0	
Missing	51		12.65%		542		15.75%		0 **	593 (15.43%) ***
**Health/** **Studies-M**	**Primary**		**Gymnasium**		**High school**		**Academic**		**Missing**	p *
	**No.**	**%**	**No.**	**%**	**No.**	**%**	**No.**	**%**		
Poor	3	5.6%	12	9.2%	21	1.9%	11	0.6%	7 (12.96%)	<0.001
Satisfactory	4	7.4%	13	9.9%	104	9.2%	74	4.3%	21 (9.72%)	
Good	31	57.4%	57	43.5%	513	45.6%	560	32.7%	89 (7.12%)	
Very good	12	22.2%	35	26.7%	340	30.2%	723	42.1%	79 (6.64%)	
Excellent	4	7.4%	14	10.7%	148	13.1%	349	20.3%	26 (4.8%)	
Missing	33	37.93%	31	19.13%	168	12.98%	145	7.78%	216 (5.62%) **	815 (21.2%) ***
**Health/** **Studies-F**	**Primary**		**Gymnasium**		**High school**		**Academic**		**Missing**	p *
	**No.**	**%**	**No.**	**%**	**No.**	**%**	**No.**	**%**		
Poor	3	4.7%	12	9.7%	22	2.4%	15	0.7%	2 (3.7%)	<0.001
Satisfactory	3	4.7%	17	13.7%	90	9.7%	102	4.9%	4 (1.85%)	
Good	35	54.6%	52	41.9%	454	49%	686	33.1%	23 (1.84%)	
Very good	17	26.6%	35	28.2%	249	26.9%	866	41.7%	22 (1.85%)	
Excellent	6	9.4%	8	6.5%	111	12%	408	19.6%	8 (1.47%)	
Missing	23	26.43%	52	29.54%	152	14.1%	193	8.5%	173 (4.5%) **	652 (16.96%) ***

* Fisher’s Exact Test, ** Missing data with none of the characteristics observed, *** Total missing.

**Table 3:** Distribution of participants related to the parental socioeconomic status and frequency of dental hygiene among children.

**Hygiene Frequency**	**Rural**				**Urban**					***p***
	**No.**		**%**		**No.**		**%**		**Missing**	
Never	1		0.3%		7		0.2%		0	0.563
A few times a month	2		0.5%		27		0.8%		0	
Once a week	5		1.3%		50		1.5%		0	
Several times a week	26		6.6%		266		8.2%		0	
Once a day	166		42.3%		1231		37.9%		0	
Two or more times a day	192		49%		1666		51.4%		0	
Missing	11		2.73%		193		5.61%		0 **	204 (5.3%) ***
**Hygiene frequency/** **Studies-M**	**Primary**		**Gymnasium**		**High school**		**Academic**		**Missing**	p *
	**No.**	**%**	**No.**	**%**	**No.**	**%**	**No.**	**%**		
Never	2	2.3%	0	0%	2	0.2%	1	0.1%	3 (37.5%)	<0.001
A few times a month	14	16.3%	1	0.6%	11	0.9%	0	0%	3 (10.34%)	
Once a week	8	9.3%	10	6.3%	21	1.6%	13	0.7%	3 (5.45%)	
Several times a week	12	14%	27	17.1%	106	8.3%	121	6.6%	26 (8.9%)	
Once a day	35	40.7%	73	46.3%	550	43%	622	33.7%	117 (8.37%)	
Two or more times a day	15	17.4%	47	29.7%	590	46%	1087	58.9%	119 (6.4%)	
Missing	1	1.15%	4	2.47%	14	1.08%	18	0.96%	167 (4.34%) **	475 (12.36%) ***
**Hygiene frequency/** **Studies-F**	**Primary**		**Gymnasium**		**High school**		**Academic**		**Missing**	p *
	**No.**	**%**	**No.**	**%**	**No.**	**%**	**No.**	**%**		
Never	2	2.3%	1	0.6%	3	0.3%	1	0%	1 (12.5%)	<0.001
A few times a month	11	12.8%	9	5.2%	7	0.7%	2	0.1%	0 (0%)	
Once a week	10	11.6%	10	5.8%	17	1.5%	15	0.7%	3 (5.45%)	
Several times a week	13	15.1%	20	11.6%	95	9%	158	7%	6 (2.05%)	
Once a day	31	36%	76	43.9%	469	44.3%	793	35.3%	28 (2%)	
Two or more times a day	19	22.2%	57	32.9%	468	44.2%	1279	56.9%	35 (1.88%)	
Missing	1	1.15%	3	1.7%	19	1.76%	22	0.97%	159 (4.13%) **	277 (7.2%) ***

* Fisher’s Exact Test, ** Missing data with none of the characteristics observed, *** Total missing.

**Table 4:** Distribution of participants regarding the use of toothpaste/fluoride toothpaste.

**Use of Toothpaste**	**Rural**				**Urban**					***p***
	**No.**		**%**		**No.**		**%**		**Missing**	
Absent	0		0%		29		0.9%		0	0.067
Present	393		100%		3229		99.1%		0	
Missing	10		2.48%		182		5.29%		0 **	192 (5%) ***
**Use of toothpaste** **/Studies-M**	**Primary**		**Gymnasium**		**High school**		**Academic**		**Missing**	p *
	**No.**	**%**	**No.**	**%**	**No.**	**%**	**No.**	**%**		
Absent	12	14%	3	1.9%	8	0.6%	4	0.2%	2 (6.9%)	<0.001
Present	74	86%	156	98.1%	1274	99.4%	1844	99.8%	274 (7.56%)	
Missing	1	1.15%	3	1.85%	12	0.92%	14	0.75%	162 (4.21%) **	468 (12.1%) ***
**Use of toothpaste** **/Studies-F**	**Primary**		**Gymnasium**		**High school**		**Academic**		**Missing**	p *
	**No.**	**%**	**No.**	**%**	**No.**	**%**	**No.**	**%**		
Absent	12	14.3%	3	1.7%	7	0.7%	7	0.3%	0 (0%)	<0.001
Present	72	85.7%	170	98.3%	1060	99.3%	2248	99.7%	72 (1.98%)	
Missing	3	3.44%	3	1.7%	11	1.02%	15	0.66%	160 (4.16%) **	264 (6.87%) ***
**Use of fluoride toothpaste/Environment**	**Rural**				**Urban**				**Missing**	***p*** *
	**No.**		**%**		**No.**		**%**			
Absent	88		26.3%		634		22.7%		0	0.131
Present	246		73.7%		2165		77.3%		0	
Missing	69		17.12%		641		18.63%		0 **	710 (18.4%) ***
**Use of fluoride toothpaste** **/Studies-M**	**Primary**		**Gymnasium**		**High school**		**Academic**		**Missing**	p *
	**No.**	**%**	**No.**	**%**	**No.**	**%**	**No.**	**%**		
Absent	32	58.2%	37	31.1%	264	24.8%	334	20.1%	55 (7.61%)	<0.001
Present	23	41.8%	82	68.9%	801	75.2%	1329	79.9%	176 (7.3%)	
Missing	32	36.78%	43	26.54%	229	17.7%	199	10.7%	207 (5.38%) **	941 (24.5%) ***
**Use of fluoride toothpaste** **/Studies-F**	**Primary**		**Gymnasium**		**High school**		**Academic**		**Missing**	p *
	**No.**	**%**	**No.**	**%**	**No.**	**%**	**No.**	**%**		
Absent	30	57.7%	41	31.5%	240	27.2%	399	19.9%	12 (1.66%)	<0.001
Present	22	42.3%	89	68.5%	641	72.8%	1609	80.1%	50 (2.07%)	
Missing	35	40.22%	46	26.13%	197	18.3%	262	11.5%	170 (4.42%) **	772 (20%) ***

* Fisher’s Exact Test, ** Missing data with none of the characteristics observed, *** Total missing.

**Table 5:** Distribution of participants related to parents’ socioeconomic status and use of brushing aids.

**Use of Toothbrush**	**Rural**				**Urban**					***p***
	**No.**		**%**		**No.**		**%**		**Missing**	
Absent	1		0.3%		20		0.6%		0	0.720
Present	386		99.7%		3198		99.4%		0	
Missing	16		3.97%		222		6.45%		0 **	238 (6.2%) ***
**Use of toothbrush** **/Studies-M**	**Primary**		**Gymnasium**		**High school**		**Academic**		**Missing**	***p***
	**No.**	**%**	**No.**	**%**	**No.**	**%**	**No.**	**%**		
Absent	1	1.2%	0	0%	9	0.7%	9	0.5%	2 (9.52%)	0.416
Present	83	98.8%	162	100%	1264	99.3%	1812	99.5%	263 (7.33%)	
Missing	3	3.44%	0	0%	21	1.62%	41	2.2%	173 (4.5%) **	503 (13%) ***
**Use of toothbrush** **/Studies-F**	**Primary**		**Gymnasium**		**High school**		**Academic**		**Missing**	p *
	**No.**	**%**	**No.**	**%**	**No.**	**%**	**No.**	**%**		
Absent	4	4.7%	0	0%	9	0.9%	8	0.4%	0 (0%)	0.001
Present	81	95.3%	174	100%	1042	99.1%	2215	99.6%	72 (2%)	
Missing	2	2.3%	2	1.13%	27	2.5%	47	2.07%	160 (4.16%) **	310 (8.06%) ***
**Dental floss use** **/Environment**	**Rural**				**Urban**				**Missing**	*p* *
	**No.**		**%**		**No.**		**%**			
Absent	309		79.8%		2539		78.9%		0	0.692
Present	78		20.2%		679		21.1%		0	
Missing	16		3.97%		222		6.45%		0 **	238 (6.2%) ***
**Dental floss use** **/Studies-M**	**Primary**		**Gymnasium**		**High school**		**Academic**		**Missing**	*p* *
	**No.**	**%**	**No.**	**%**	**No.**	**%**	**No.**	**%**		
Absent	83	98.8%	152	93.8%	1074	84.4%	1322	72.6%	217 (7.62%)	<0.001
Present	1	1.2%	10	6.2%	199	15.6%	499	27.4%	48 (6.34%)	
Missing	3	3.44%	0	0%	21	1.62%	41	2.2%	173 (4.5%) **	503 (13%) ***
**Dental floss use** **/Studies-F**	**Primary**		**Gymnasium**		**High school**		**Academic**		**Missing**	*p* *
	**No.**	**%**	**No.**	**%**	**No.**	**%**	**No.**	**%**		
Absent	82	96.5%	159	91.4%	909	86.5%	1636	73.6%	62 (2.17%)	<0.001
Present	3	3.5%	15	8.6%	142	13.5%	587	26.4%	10 (1.32%)	
Missing	2	2.3%	2	1.13%	27	2.5%	47	2.07%	160 (4.16%) **	310 (8.06%) ***
**Use of wooden toothpicks** **/Environment**	**Rural**				**Urban**				**Missing**	***p***
	**No.**		**%**		**No.**		**%**			
Absent	342		88.4%		2883		89.6%		0	0.483
Present	45		11.6%		335		10.4%		0	
Missing	16		3.97%		222		6.45%		0 **	238 (6.2%) ***
**Use of wooden toothpicks** **/Studies-M**	**Primary**		**Gymnasium**		**High school**		**Academic**		**Missing**	***p***
	**No.**	**%**	**No.**	**%**	**No.**	**%**	**No.**	**%**		
Absent	79	94%	150	92.6%	1118	87.8%	1641	90.1%	237 (7.34%)	0.052
Present	5	6%	12	7.4%	155	12.2%	180	9.9%	28 (7.36%)	
Missing	3	3.44%	0	0%	21	1.62%	41	2.2%	173 (4.5%) **	503 (13%) ***
**Use of wooden toothpicks** **/Studies-F**	**Primary**		**Gymnasium**		**High school**		**Academic**		**Missing**	***p***
	**No.**	**%**	**No.**	**%**	**No.**	**%**	**No.**	**%**		
Absent	79	92.9%	162	93.1%	929	88.4%	1990	89.5%	65 (2.01%)	0.199
Present	6	7.1%	12	6.9%	122	11.6%	233	10.5%	7 (1.84%)	
Missing	2	2.3%	2	1.13%	27	2.5%	47	2.07%	160 (4.16%) **	310 (8.06%) ***
**Use of plastic toothpicks** **/Environment**	**Rural**				**Urban**				**Missing**	***p***
	**No.**		**%**		**No.**		**%**			
Absent	378		97.7%		3135		97.4%		0	0.866
Present	9		2.3%		83		2.6%		0	
Missing	16		3.97%		222		6.45%		0 **	238 (6.2%) ***
**Use of plastic toothpicks** **/Studies-M**	**Primary**		**Gymnasium**		**High school**		**Academic**		**Missing**	p *
	**No.**	**%**	**No.**	**%**	**No.**	**%**	**No.**	**%**		
Absent	84	100%	160	98.8%	1252	98.4%	1763	96.8%	254 (7.23%)	0.015
Present	0	0%	2	1.2%	21	1.6%	58	3.2%	11 (11.95%)	
Missing	3	3.44%	0	0%	21	1.62%	41	2.2%	173 (4.5%) **	503 (13%) ***
**Use of plastic toothpicks** **/Studies-F**	**Primary**		**Gymnasium**		**High school**		**Academic**		**Missing**	p *
	**No.**	**%**	**No.**	**%**	**No.**	**%**	**No.**	**%**		
Absent	84	98.8%	172	98.9%	1037	98.7%	2150	96.7%	70 (2%)	0.004
Present	1	1.2%	2	1.1%	14	1.3%	73	3.3%	2 (2.17%)	
Missing	2	2.3%	2	1.13%	27	2.5%	47	2.07%	160 (4.16%) **	310 (8.06%) ***

* Fisher’s Exact Test, ** Missing data with none of the characteristics observed, *** Total missing.

**Table 6:** Multinomial and binomial logistic regression models used in predicting effects of parents’ studies across children’s oral health.

Parameter		Univariable		Multivariable	
Children teeth health—perceived status					
Teeth status/Parent studies		OR (95% C.I.)	*p*	OR (95% C.I.)	*p*
Poor (Reference)	Academic-M	-	-	-	-
	Academic-F	-	-	-	-
Satisfactory	Academic-M	1.855 (1.256–2.74)	0.002	1.172 (0.712–1.931)	0.533
	Academic-F	2.232 (1.563–3.195)	<0.001	2.02 (1.25–3.268)	0.004
Good	Academic-M	2.571 (1.808–3.65)	<0.001	1.721 (1.096–2.695)	0.018
	Academic-F	2.625 (1.908–3.61)	<0.001	1.838 (1.196–2.825)	0.005
Very good	Academic-M	4.31 (2.994–6.173)	<0.001	2.179 (1.372–3.46)	0.001
	Academic-F	5 (3.571–6.993)	<0.001	2.976 (1.898–4.651)	<0.001
Excellent	Academic-M	5.464 (3.584–8.333)	<0.001	3.077 (1.792–5.291)	<0.001
	Academic-F	5.587 (3.704–8.403)	<0.001	2.611 (1.52–4.484)	0.001
Children Gums health—perceived status					
Gums status/Parent studies		OR (95% C.I.)	*p*	OR (95% C.I.)	*p*
Poor (Reference)	Academic-M	-	-	-	-
	Academic-F	-	-	-	-
Satisfactory	Academic-M	2 (0.96–4.167)	0.064	0.984 (0.39–2.481)	0.972
	Academic-F	2.288 (1.185–4.425)	0.014	2.941 (1.215–7.194)	0.018
Good	Academic-M	3.049 (1.536–6.061)	0.001	1.572 (0.665–3.717)	0.302
	Academic-F	3.125 (1.698–5.747)	<0.001	2.809 (1.217–6.494)	0.016
Very good	Academic-M	6.098 (3.077–12.195)	<0.001	2.123 (0.895–5.025)	0.087
	Academic-F	7.092 (3.846–13.158)	<0.001	5.464 (2.364–12.658)	<0.001
Excellent	Academic-M	6.897 (3.413–13.889)	<0.001	2.392 (0.993–5.78)	0.052
	Academic-F	8.065 (4.274–15.152)	<0.001	5.682 (2.398–13.514)	<0.001
Children oral hyiene habits—frequency of toothbrushing					
Frequency/Parent studies		OR (95% C.I.)	*p*	OR (95% C.I.)	*p*
Never/A few times a month (Reference)	Academic-M	-	-	-	-
	Academic-F	-	-	-	-
Once a week	Academic-M	10 (1.238–83.333)	0.031	7.463 (0.762–71.429)	0.084
	Academic-F	4.464 (1.185–16.667)	0.027	1.818 (0.408–8.065)	0.433
Several times a week	Academic-M	25 (3.367–200)	0.002	9.524 (1.103–83.333)	0.040
	Academic-F	13.514 (4.065–45.455)	<0.001	4.566 (1.23–16.949)	0.023
Once a day	Academic-M	28.571 (3.861–200)	0.001	10.309 (1.225–90.91)	0.032
	Academic-F	15.152 (4.63–50)	<0.001	4.808 (1.332–17.241)	0.016
Two or more times a day	Academic-M	50 (6.803–333.33)	<0.001	15.873 (1.873–142.857)	0.011
	Academic-F	25.641 (7.874–83.333)	<0.001	6.098 (1.689–21.739)	0.006
Children oral hyiene habits—usage of toothpaste					
Parent studies		OR (95% C.I.)	*p*	OR (95% C.I.)	*p*
Academic-M		7.05 (2.433–20.429)	<0.001	3.405 (0.943–12.295)	0.061
Academic-F		5.426 (2.312–12.737)	<0.001	3.063 (1.021-9.186)	0.046
Children oral hyiene habits—usage of fluoride toothpaste					
Parent studies		OR (95% C.I.)	*p*	OR (95% C.I.)	*p*
Academic-M		1.462 (1.229–1.74)	<0.001	1.130 (0.899–1.421)	0.296
Academic-F		1.668 (1.405–1.98)	<0.001	1.484 (1.174–1.875)	0.001
Children oral hyiene habits—usage of toothbrush					
Parent studies		OR (95% C.I.)	*p*	OR (95% C.I.)	*p*
Academic-M		1.186 (0.456–3.084)	0.726	-	-
Academic-F		2.379 (0.936–6.045)	0.069	-	-
Children oral hyiene habits—usage of dental floss					
Parent studies		OR (95% C.I.)	*p*	OR (95% C.I.)	*p*
Academic-M		2.379 (1.96–2.887)	<0.001	1.62 (1.271–2.064)	<0.001
Academic-F		2.751 (2.225–3.401)	<0.001	1.964 (1.497–2.577)	<0.001
Children oral hyiene habits—usage of plastic toothpicks					
Parent studies		OR (95% C.I.)	*p*	OR (95% C.I.)	*p*
Academic-M		2.212 (1.306–3.747)	0.003	1.381 (0.735–2.597)	0.316
Academic-F		2.87 (1.579–5.217)	0.001	2.407 (1.112–5.21)	0.026

## Data Availability

The data presented in this study are available from the corresponding authors upon reasonable request.
